# Impact of Morphine Dependence and Withdrawal on the Reinforcing Effectiveness of Fentanyl, Cocaine, and Methamphetamine in Rats

**DOI:** 10.3389/fphar.2021.691700

**Published:** 2021-05-21

**Authors:** Robert W. Seaman Jr, Gregory T. Collins

**Affiliations:** ^1^ Department of Pharmacology, The University of Texas Health Science Center at San Antonio, San Antonio, TX, United States; ^2^ South Texas Veterans Health Care System, San Antonio, TX, United States

**Keywords:** withdrawal, polysubstance abuse, opioids, stimulants, progressive ratio

## Abstract

Recent estimates suggest increased popularity of the concurrent use of opioids and stimulants, with over 50% of treatment-seeking opioid users reporting regular stimulant use. The goal of the current study was to determine how opioid dependence and withdrawal affect the reinforcing effects of fentanyl, cocaine, and methamphetamine. Male Sprague-Dawley rats were allowed to self-administer fentanyl under a progressive ratio (PR) schedule of reinforcement. Baseline evaluations of reinforcing effectiveness of fentanyl, cocaine, and methamphetamine were determined. Opioid dependence was then established by administering escalating doses of morphine (10–40 mg/kg) twice-daily for four days and subsequently maintained by once-daily injections of 40 mg/kg morphine. To evaluate the impact of opioid dependence and withdrawal on the self-administration of fentanyl, cocaine, and methamphetamine, sessions occurred either 12 or 20 h after the morphine, respectively. During opioid withdrawal, the fentanyl dose-response curve was shifted rightward with an increase in maximal effectiveness, whereas it was shifted rightward with a reduction in maximal effectiveness when evaluated in rats currently dependent on opioids, relative to baseline. The reinforcing effects of cocaine and methamphetamine were unchanged by either condition. The current studies provide direct evidence that the reinforcing effects of fentanyl are increased in opioid-withdrawn rats and reduced in opioid-dependent rats, relative to rats that are not physically dependent on opioids. These findings suggest that motivations to use opioids are dependent on the state of the individual whereas stimulants retain their reinforcing effects regardless of whether the individual is in an opioid-dependent or withdrawn state.

## Introduction

The opioid crisis continues to pose a significant health burden, as evidenced by approximately 50 million people reported to have misused opioids worldwide in 2018 (United Nations Office on Drugs and Crime, 2019). Coinciding with the high incidence of opioid abuse is the increased prevalence of ultra-potent μ-opioid receptor agonists, such as fentanyl, in opioid supply, a trend that was responsible for more than 36,000 overdose deaths in the United States during 2019, or ∼12-times more than occurred in 2013 (Mattson et al., 2021). One major hurdle in treating opioid use disorder and preventing opioid-induced overdose is the relatively high rate of relapse following a period of abstinence, with one study reporting that ∼60% of individuals relapse in the first week of abstinence and ∼80% relapse in the first month (Smyth et al., 2010). One proposed contributor to the relatively high relapse rates in individuals with opioid use disorder is the ability of opioids to reverse/terminate symptoms associated with withdrawal from opioids (Solomon and Corbit, 1974; Koob and Le Moal, 2001; Colpaert et al., 2006; Koob, 2020). The cessation of opioid use in an individual that is physically dependent on opioids results in the emergence of a withdrawal syndrome comprising of psychological (e.g., anxiety, craving) and physiological (e.g., nausea, diarrhea) symptoms with varying intensities which together can culminate in a highly aversive state for the individual. While the reinforcing effects of fentanyl have been previously characterized in rats (Lal et al., 1977; van Ree et al., 1978; Awasaki et al., 1997; Morgan et al., 2002; Wade et al., 2015; Wager et al., 2017), much less is known about how opioid dependence and withdrawal impact the reinforcing effects of fentanyl (Townsend et al., 2019), or other commonly co-abused drugs.

The abuse of multiple substances, particularly stimulants and opioids, is not a new phenomenon, and there is increasing awareness among treatment providers, policy makers, and basic scientists that polysubstance abuse is the norm rather than the exception (for a review, see (Crummy et al., 2020)). Indeed, one report found that individuals classified as, “drug-dependent” use an average of 3.5 substances, including both simultaneous and sequential polydrug use (Onyeka et al., 2012). Several studies put the share of treatment-seeking opioid users who are also current users of stimulants (e.g., cocaine, methamphetamine, cathinones) at over 50% (Kidorf et al., 2018; Lee et al., 2018). Moreover, state-level and national estimates suggest that ∼50% of individuals who overdose on opioids were also using stimulants at the time of their death (DuPont, 2018; Jones et al., 2018; Avedschmidt et al., 2019; Colon-Berezin et al., 2019). Furthermore, from 2014 through 2018, the rate of drug overdose deaths involving cocaine with concurrent involvement of opioids increased at a faster pace than drug overdose deaths involving cocaine without opioid involvement (Hedegaard et al., 2020). Historically, co-use of stimulants and opioids has largely been one-sided (e.g., opioid users adding cocaine to their heroin), but recent evidence suggests that this trend may be shifting, with increasing and widespread reports of opioids, primarily fentanyl, being mixed with both cocaine and methamphetamine (Jones et al., 2018; DEA-MIA-BUL-039-18).

Contrasting societal prevalence, few preclinical studies, particularly using rodent models, have evaluated the relationship between opioid withdrawal and the reinforcing effects of opioids and/or stimulants (Cooper et al., 2008; Cooper et al., 2010; Townsend et al., 2019). One procedure that has been particularly useful in studying these relationships involves escalating, twice-daily doses of morphine (10–40 mg/kg) to establish physical dependence, followed by once daily dosing with 40 mg/kg morphine to maintain rats in a morphine-dependent state, followed by the emergence of somatic signs of withdrawal ∼18 h later. This dosing regimen has been shown to produce a consistent, daily withdrawal syndrome in rats that allows for longitudinal studies of how opioid withdrawal impacts the reinforcing effects of opioids (Houshyar et al., 2003; Cooper et al., 2008; Cooper et al., 2010). For instance, rats allowed to self-administer the short-acting μ-opioid receptor agonist, remifentanil, under a fixed ratio schedule of reinforcement ∼20 h after their last dose of morphine self-administered more remifentanil than both rats that were deprived of morphine for ∼12 h, as well as rats that were not dependent on morphine (Cooper et al., 2008; Cooper et al., 2010) suggesting that morphine withdrawal can impact the reinforcing effects of opioids under some conditions. Since responding maintained under fixed ratio schedules of reinforcement can be influenced by the pharmacokinetic and pharmacodynamic properties of drugs, these schedules are not ideal to evaluate reinforcing effectiveness (Griffiths et al., 1979; Katz, 1990; Roberts, 1993; Richardson and Roberts, 1996; Ko et al., 2002). To this end, the current study utilized the same regimen of noncontingent morphine dosing paired with self-administration under a progressive ratio (PR) schedule of reinforcement to test the hypotheses that: 1) morphine-dependent rats will exhibit increased levels of somatic withdrawal signs and sensitivity to a mechanical stimulus following 20, but not 12 h of morphine deprivation; 2) the reinforcing effectiveness of fentanyl will be reduced after 12 h of morphine deprivation, but enhanced after 20 h of morphine deprivation, relative to a non-dependent baseline evaluation; and 3) the reinforcing effectiveness of methamphetamine and cocaine will be unchanged regardless of morphine withdrawal state, relative to a non-dependent baseline evaluation.

## Methods

**Subjects:** Twelve male Sprague-Dawley rats (275–300 g upon arrival) were purchased from Envigo (Indianapolis, IN, United States) and maintained in a temperature- and humidity-controlled room. Rats were individually housed and maintained on a 14/10 h light/dark cycle. All experiments were conducted during the light cycle with sessions conducted at approximately the same time each morning. Rats were provided *ad libitum* access to Purina rat chow and water. All studies were carried out in accordance with the Institutional Animal Care and Use Committees of the University of Texas Health Science Center at San Antonio and the eighth edition of the Guide for Care and Use of Laboratory Animals (National Research Council (United States) Committee for the Update of the Guide for the Care and Use of Laboratory Animals 2011).

**Surgery:** Rats were anesthetized with 2–3% isoflurane and prepared with chronic indwelling catheters in the left femoral vein, as previously described (Gannon et al., 2017; Gannon et al., 2018). Catheters were tunneled under the skin and attached to a vascular access button placed in the mid-scapular region. Immediately following surgery, rats were administered Penicillin G (60,000 U/rat) subcutaneously to prevent infection. Rats were allowed 5–7 days to recover during which time catheters were flushed daily with 0.5 ml of heparinized saline (100 U/ml). Thereafter, catheters were flushed daily with 0.2 ml of saline prior to and 0.5 ml of heparinized saline after the completion of self-administration sessions.

**Drugs:** Morphine, fentanyl, and cocaine were provided by the National Institute on Drug Abuse Drug Supply Program. D-methamphetamine was purchased from Sigma-Aldrich (St. Louis, MO, United States). All drugs were dissolved in physiological saline. To induce and maintain morphine dependence, morphine was administered via subcutaneous injections in a volume of 1.0 ml/kg. For self-administration studies, infusions were delivered intravenously in a volume of 0.1 ml/kg over ∼1 s.

**Apparatus:** All experiments were conducted in standard operant conditioning chambers located within ventilated, sound-attenuating enclosures (Med Associates, Inc., St. Albans, VT). Each chamber was equipped with two response levers located 6.8 cm above the grid floor and 1.3 cm from the right or left wall. Visual stimuli were provided by two sets of green, yellow, and red LEDs, one set located above each of the two levers, and a white house light located at the top center of the opposite wall. Drug solutions were delivered by a variable speed syringe pump through Tygon tubing connected to a stainless-steel fluid swivel and spring tether, which was held in place by a counterbalanced arm. Experimental events were controlled, and data were collected using MED-PC IV software and a PC-compatible interface (Med Associates, Inc.).

## Self-Administration

**Acquisition:** Rats were initially allowed to respond for 0.0032 mg/kg/infusion fentanyl under a fixed ratio (FR) 1 schedule of reinforcement during at least 10, daily, 90 min sessions. Illumination of a yellow LED above the active lever (left or right; counterbalanced across rats) signaled drug availability and completion of the response requirement resulted in a drug infusion (0.1 ml/kg over ∼1 s), illumination of the houselight as well as the yellow, green, and red LEDs above the active lever, and initiation of a 5 s timeout throughout which drug was made unavailable. The infusion-paired stimuli were present throughout the 5 s timeout. Responses on the inactive lever, as well as responses on either lever during timeouts, were recorded but had no scheduled consequences. Acquisition criteria were as follows: ≥15 infusions for two consecutive days with ≥80% responding occurring on the active lever vs. the inactive lever. Response requirements were subsequently increased to an FR 5 schedule where they remained until stability criteria were met (±20% of the mean number of infusions for three consecutive sessions and no increasing or decreasing trend).

**Progressive ratio dose-response curves:** After reaching stability under an FR 5 schedule, rats were transitioned to a progressive ratio (PR) schedule of reinforcement under which the ratios were incremented after each subsequent infusion according to the following equation: response requirement = [5e^(infusion number × 0.2)] − 5 (Richardson and Roberts, 1996). Sessions lasted until a ratio was not completed within 45 min (i.e., 45 min limited hold). Ratio completion resulted in delivery of a unit dose of fentanyl (0.000032–0.01 mg/kg/infusion), cocaine (0.32 mg/kg/infusion), methamphetamine (0.032 mg/kg/infusion), or saline coinciding with illumination of the houselight as well as the yellow, green, and red LEDs above the active lever, and a 5 s timeout. Fentanyl was always evaluated first, followed by cocaine or methamphetamine (counter-balanced across rats). All doses of a particular drug were evaluated in random order before moving to the next drug. Stability was defined as two consecutive sessions where the number of infusions obtained differed by ≤ 2. The fentanyl dose-response curve, as well as responding maintained by cocaine and methamphetamine, were redetermined twice, once in rats following 12 h morphine deprivation, and then again in rats following 20 h morphine deprivation. Saline substitution was performed regularly throughout the experiment. The initial training dose of 0.0032 mg/kg/inf of fentanyl was doubly determined in all rats under all conditions and did not differ significantly between first and second determinations.

**Induction of opioid dependence:** Upon completion of baseline determinations of the reinforcing effectiveness of fentanyl, methamphetamine, and cocaine, rats were made morphine-dependent using the dosing regimen described above (Houshyar et al., 2003; Cooper et al., 2008; Cooper et al., 2010). Briefly, rats were treated with escalating doses of morphine (10, 20, 30, and 40 mg/kg; subcutaneously) twice daily (every 12 h) for four days. Beginning on the fifth day, half of the rats were given saline in the morning and the maintenance dose of 40 mg/kg morphine in the evening (morphine dependent; MD). The other half of the rats continued to receive the maintenance dose of morphine in the morning and received saline in the evening (morphine withdrawn; MW). Self-administration sessions commenced the morning of the sixth day after initiation of the injection schedule for both groups of animals (zeitgeber time = 2). The MW group received morphine approximately 30 min after the completion of the self-administration session whereas the MD group received 40 mg/kg morphine in the evening, ∼12 h prior to the start of the self-administration session. Once the dose-response curve for fentanyl was redetermined along with 0.032 and 0.32 mg/kg/infusion unit doses of methamphetamine and cocaine, respectively, conditions were switched (i.e., MW group became MD group and vice-versa). This allowed for the reinforcing effectiveness of fentanyl, methamphetamine, and cocaine to be evaluated prior to induction of dependence, in an opioid-dependent state, and in an opioid-withdrawn state in a within-subject design. The order of testing was counter-balanced, and no differences were observed in the fentanyl dose-response curves, or cocaine and methamphetamine breakpoints, as a function of testing order. In the event that catheter patency was lost prior to the end of the study, the rat was removed from the study and did not contribute any further data (i.e., only data collected prior to this point was included for analyses).

**Quantification of opioid withdrawal:** Rats were placed on a wire mesh stand in plastic enclosures (Model 435; IITC Inc. Life Science) and were observed for somatic withdrawal signs including wet dog shakes, ptosis, paw tremors, teeth chattering, teeth grinding, and diarrhea. Somatic withdrawal signs were quantified across three, 5 min blocks, with each block separated by 10 min. Directly following this observation period, sensitivity to a mechanical stimulus was evaluated using an electronic Von Frey probe with a rigid tip (Models 2,396 and 2,391; IITC Inc. Life Science) by measuring paw-withdrawal threshold in grams of force. Measurements of withdrawal and mechanical nociception were taken prior to the induction of dependence, on the sixth day after induction of dependence, and then 72 h after the conditions were switched.

**Change in body weight:** Every 12 h (±2 h), rats were weighed before injections (either saline or morphine). Recorded weights from the experimental period were subtracted from baseline weights for individual rats, defined as the average body weight for the 7 days prior to the induction of opioid dependence.

## Data Analysis

**Change in body weight:** Data are presented as the mean ± SEM change in body weight from baseline. A two-way, mixed-methods repeated-measures analysis of variance (ANOVA) was used to determine the main effects of morphine dependence and time on weight change.

**Quantification of opioid withdrawal and mechanical nociception:** Data are presented as the mean ± SEM of the number of withdrawal signs noted in the observation periods. A one-way, mixed-methods repeated-measures ANOVA with post-hoc Tukey’s tests was used to determine whether the number of withdrawal signs and mechanical nociception values differed as a function of the state of opioid dependence.

**Progressive ratio:** Data are presented as the mean ± SEM of the number of infusions obtained at each unit dose. For each subject, the greatest number of infusions earned for fentanyl (i.e., Emax) was identified, with mean Emax values (±SEM) serving as a dose-independent measure of relative reinforcing effectiveness. A one-way, mixed-methods repeated-measures ANOVA with post-hoc Dunnet’s tests were used to determine if Emax values for fentanyl, as well as the number of cocaine, methamphetamine, and saline infusions, differed as a function of the state of opioid dependence (i.e., non-dependent, MD, MW).

## Results

**FIGURE 1:**
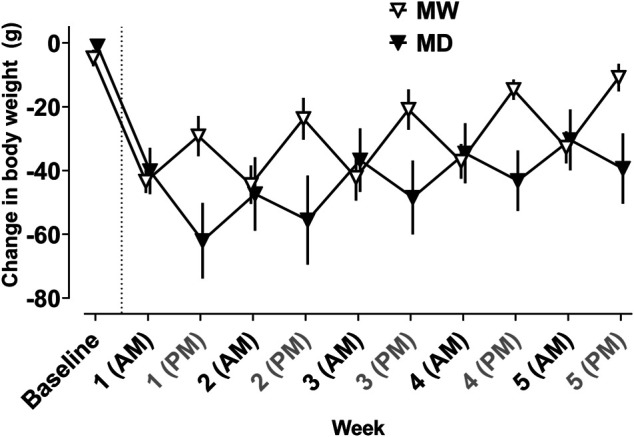
Average change in body weight (g) from baseline weight measured every 12 h in the 12 h (MD) and 20 h (MW) deprivation groups. The dotted line represents induction of dependence. All self-administration sessions occurred at AM time points. The MW group received morphine at AM time points and the MD group received morphine at PM time points. Data represent the mean (±SEM), and each point represents six to eight rats.

Figure 1 depicts changes in body weight resulting from morphine treatment. Weight gain was observed 0–12 h after the maintenance dose of morphine, whereas weight loss was observed 12–24 h after morphine treatment. A significant interaction between time and treatment condition (i.e., baseline, MD, MW) was observed (F [10,97] = 10.9; *p* < 0.001).

**FIGURE 2:**
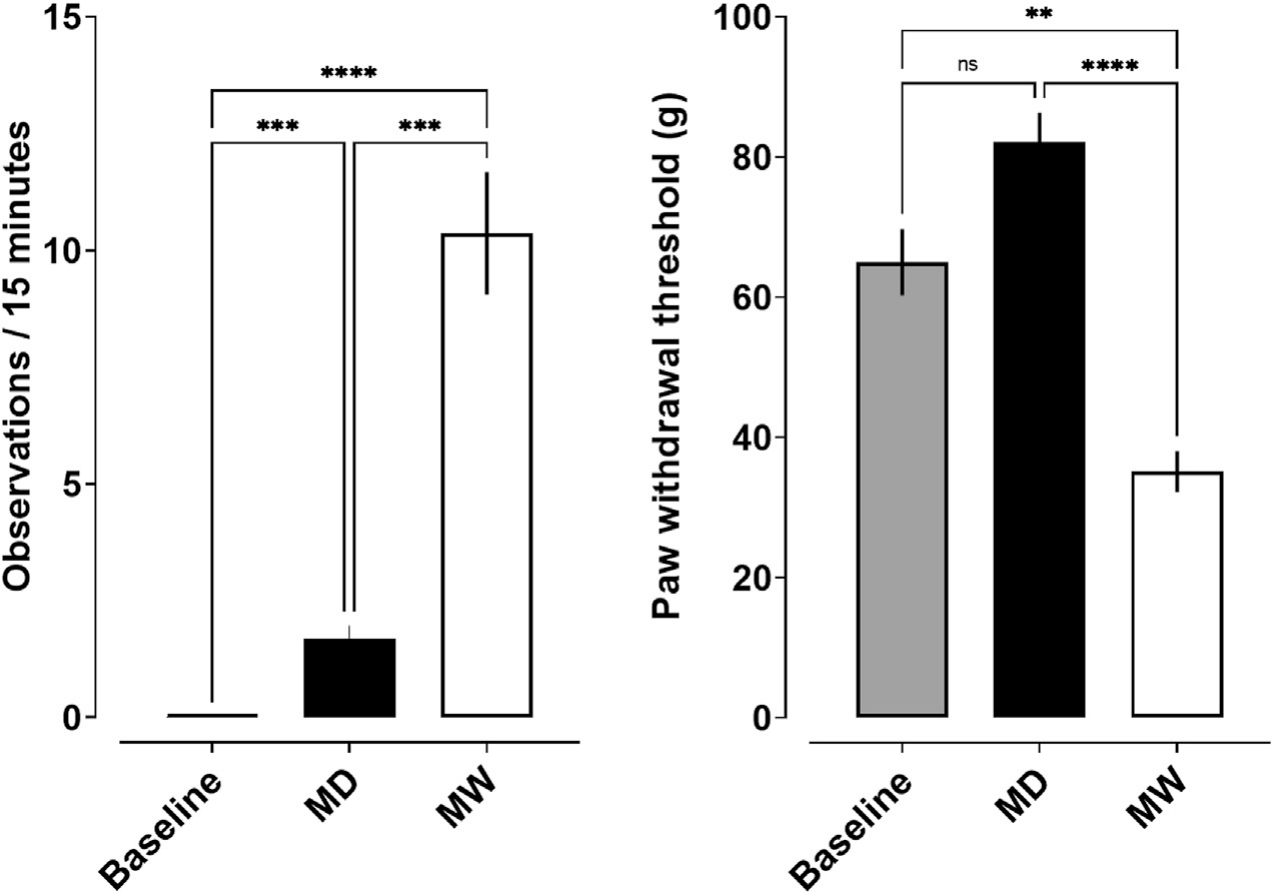
Somatic withdrawal signs **(left)** and mechanical nociception **(right)** and as a function of state of withdrawal. Data represent the mean (±SEM), and each bar represents 10–12 rats. Ns indicates not significant, **** indicates *p* < 0.0001, *** indicates *p* < 0.001, and ** indicates *p* < 0.01.

Somatic signs of opioid withdrawal were also quantified (Figure 2; left). A significant effect of treatment condition (F [1.1,10] = 53.4; *p* < 0.001) was observed. No withdrawal signs were observed prior to induction of dependence. Post-hoc tests revealed a significant increase in observable withdrawal signs in the MD group relative to baseline (1.7 ± 0.3; *p* < 0.001). The MW group exhibited a greater number of withdrawal signs (10.4 ± 1.3) than observed at both baseline and MD conditions (*p* < 0.001). Figure 2 (right) shows the impact of morphine-deprivation on mechanical nociception. A significant effect of treatment condition was observed (F [1.7,26.3] = 34.6; *p* < 0.001). Post-hoc tests revealed no significant differences in paw withdrawal threshold in the MD group (82.1 ± 4.2) relative to baseline (65 ± 4.7). However, the MW group (35.1 ± 2.9) exhibited a significantly smaller paw withdrawal threshold relative to baseline (*p* < 0.01) and the MD group (*p* < 0.001).

**FIGURE 3:**
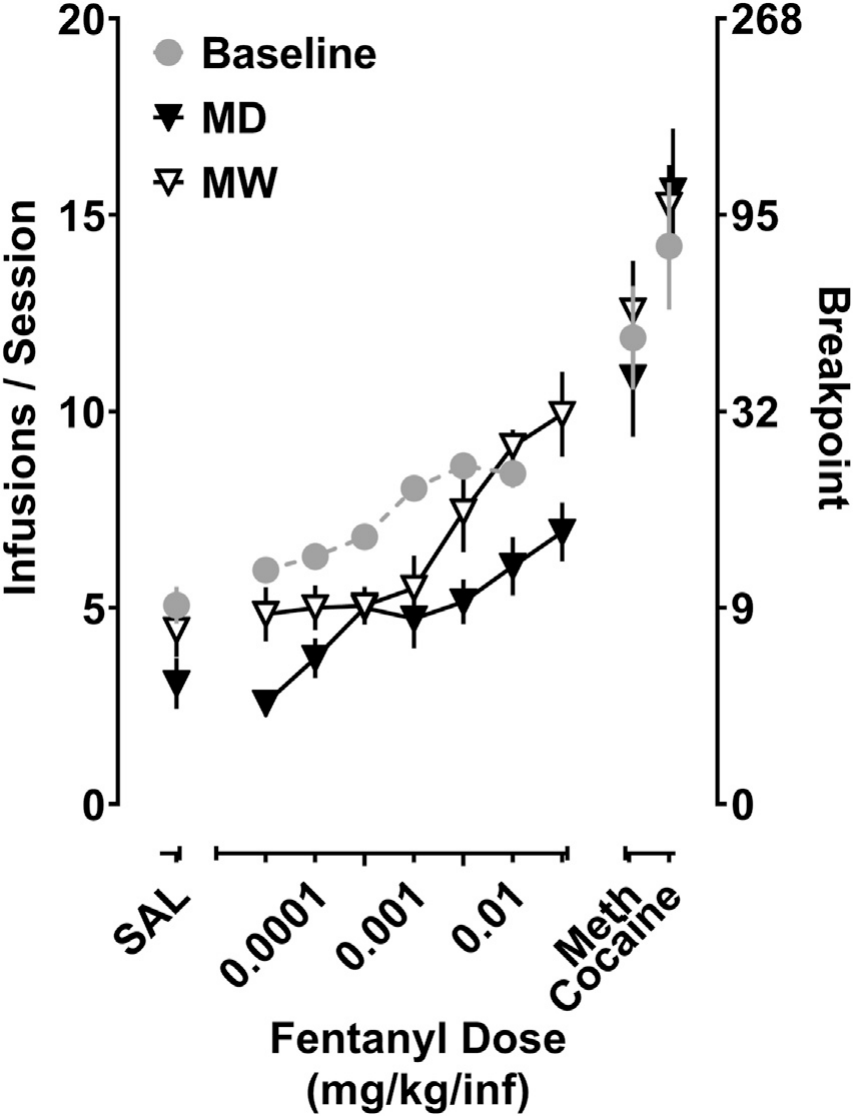
Self-administration of fentanyl, cocaine, and methamphetamine in rats responding under a progressive ratio (PR) schedule of reinforcement. Data represent the mean (±SEM) for the total number of infusions earned for fentanyl (0.0032–0.032 mg/kg/inf), cocaine (0.32 mg/kg/inf), and methamphetamine (0.032 mg/kg/inf) **(left ordinate)**, as well as the corresponding final ratio completed **(right ordinate)**. Each point represents six to eight rats.

Depicted in Figure 3 are the fentanyl dose-response curves for the total number of infusions earned for rats responding under a PR schedule of reinforcement. A significant main effect of treatment condition on Emax values (i.e., baseline, MD, MW) was observed (F [1.5,13] = 11.8; *p* < 0.01). Post-hoc tests indicate that fentanyl maintained significantly fewer infusions in the MD group (7.9 ± 0.4) relative to baseline (9.0 ± 0.3) (*p* < 0.05). In contrast, fentanyl maintained a significantly greater number of infusions in the MW group (10.4 ± 0.6), relative to baseline (*p* < 0.05). A similar relationship was observed with respect to the maximal final ratio completed (i.e., breakpoint), with the final ratios completed being smaller in the MD group (19.8 ± 1.9), and larger in the MW group (37.8 ± 4.8), relative to baseline (26.6 ± 1.9).

Regarding the reinforcing effects of cocaine (0.32 mg/kg/inf), there was no significant effect of treatment, with rats earning 14.2 ± 1.6 infusions at baseline, 15.6 ± 1.6 infusions in the MD group, and 15.2 ± 1.2 infusions in the MW group. Similarly, treatment condition had no impact on the reinforcing effectiveness of methamphetamine (0.032 mg/kg/inf), with rats earning 11.9 ± 1.3 infusions at baseline, 10.9 ± 1.5 infusions in the MD group, and 12.6 ± 1.3 infusions in the MW group. The reinforcing effectiveness of these doses of methamphetamine and cocaine resulted in peak breakpoints greater than those produced by fentanyl, under all conditions. There were no differences in the number of saline infusions earned at baseline or in the MD and MW groups, each earning 5 ± 0.5, 3.1 ± 0.6, and 4.3 ± 0.8 infusions, respectively.

## Discussion

Polysubstance abuse, often co-occurring with physical dependencies, is commonplace in clinical populations but remains largely unexplored in preclinical literature. The current studies aid in addressing this disparity through use of an intermittent, noncontingent morphine dosing regimen to determine the impact of morphine dependence and withdrawal on the reinforcing effectiveness of fentanyl, cocaine, and methamphetamine in rats. There were three main findings 1) signs of morphine withdrawal (e.g., body weight loss, hyperalgesia, somatic signs of withdrawal): were apparent following 20 h of morphine deprivation, but not 12 h, suggesting that rats cycled through states of morphine dependence and withdrawal on a daily basis; 2) morphine withdrawal produced a rightward shift in the fentanyl dose-response curve with an increase in the maximal effect, whereas morphine dependence produced a greater rightward shift with a reduction in the maximal effect of fentanyl; and 3) the reinforcing effectiveness of the doses of cocaine and methamphetamine evaluated were unaffected by states of morphine dependence and withdrawal. Taken together, these data suggest that states of opioid-dependence and withdrawal impact the reinforcing effectiveness of opioids, whereas stimulants retain their reinforcing effectiveness regardless of withdrawal state.

Consistent with previous reports (Houshyar et al., 2003; Cooper et al., 2008; Cooper et al., 2010), daily treatment with a single subcutaneous injection of 40 mg/kg morphine was sufficient to maintain rats in a state of morphine dependence, with signs of morphine withdrawal apparent by 20 h after the last dose of morphine. The current studies used several measures of morphine withdrawal to confirm that rats were physically dependent on morphine, including changes in body weight, increased sensitivity to a mechanical nociceptive stimulus, and well-characterized withdrawal-associated behaviors (e.g., wet dog shakes, ptosis, teeth chattering, diarrhea, vocalizations). These data are consistent with previous studies demonstrating that induction of morphine dependence produces a decrease in body weight that fluctuates as a function of time since prior morphine injection (Cooper et al., 2008; Cooper et al., 2010), with increases of approximately 20 g 12 h after an injection of morphine, and reductions of approximately 20 g observed by 20 h after a morphine injection. In addition to decreases in body weight, rats deprived of morphine for 20 h exhibited other signs of opioid withdrawal, including hyperalgesia and somatic signs of withdrawal. Importantly, these measures remained consistent throughout the course of the experiment. One consideration when administering morphine at different times (e.g., AM vs PM) is the impact of circadian rhythm. Although there is evidence that circadian rhythm can impact the pharmacokinetics and pharmacodynamics of morphine, this should not impact interpretations of the current data given their reliance on binary conditions of opioid dependence or withdrawal, as opposed to evaluating the effect of graded withdrawal states on reinforcing effectiveness (Miller, 1988; Kervezee et al., 2017). Taken together, these findings suggest that the regimen of morphine administration employed herein produces a stable level of morphine dependence and withdrawal, thus allowing for the impacts of such states on opioid and stimulant reinforcement to be evaluated across time.

To determine the impact that state of dependence has on the reinforcing effectiveness of fentanyl, the current self-administration studies utilized systematic, within-session increases in response requirements (i.e., progressive ratio schedule of reinforcement) to assess how hard rats would work to obtain a single infusion of fentanyl, cocaine, or methamphetamine. Consistent with previous studies, fentanyl produced dose-dependent increases in the number of infusions earned and breakpoints met (Wade et al., 2015). Both treatment conditions produced rightward shifts in the fentanyl dose-response curve. A morphine-dependent state produced a decrease in the maximal effect of fentanyl, relative to baseline, with Emax values being 7.9 and 9, respectively. In contrast, an opioid-withdrawn state produced a greater maximal effect of fentanyl, with an Emax value of 10.4. These data extend previous studies that used this regimen of morphine administration to demonstrate that 22–24 h of morphine-deprivation resulted in rats acquiring self-administration at smaller doses of the ultra-short-acting μ-opioid receptor agonist, remifentanil, and resulted in an upward shift in the remifentanil dose-response curve under a fixed ratio schedule of reinforcement, relative to non-dependent rats (Cooper et al., 2008). Conversely, Cooper and colleagues also showed that when rats tested after only 12 h of morphine deprivation, a larger dose of remifentanil was required to establish remifentanil self-administration relative to non-dependent rats. The current studies also complement previous work that utilized extended access to fentanyl self-administration to produce dependence. When evaluated in a fentanyl vs. food choice procedure, 8 h after a period of extended access self-administration (i.e., discontinuation-induced fentanyl withdrawal), the dose-response curve for fentanyl choice was shifted to the left of that obtained in non-dependent rats (Townsend et al., 2019). Differences in the effects of opioid withdrawal on the self-administration of small doses of fentanyl between the current study and those in Townsend et al. (2019) could be due to a withdrawal induced increase in fentanyl-seeking that is more apparent under low fixed ratio schedules employed in the choice procedure that are less apparent under progressive ratio schedules in which the response requirement increments with each infusion. This is supported by the significant increase in the choice of saline over food 9–13 days following the last extended access self-administration session. Additionally, although both studies relied on spontaneous opioid withdrawal, the methods of induction and maintenance of opioid dependence likely produced different degrees of opioid dependence, and therefore withdrawal syndromes of different magnitude. Taken together, these data demonstrate that deprivation-induced opioid withdrawal enhances the reinforcing effectiveness of opioids. The observed shifts in the fentanyl dose-response curves are likely the product of the complex interplay between negative reinforcement (i.e., ameliorating withdrawal), tolerance, and still-circulating morphine in rats deprived of morphine for 12 h. There are a couple of caveats to consider regarding the interpretation of the current findings. The doses of fentanyl that were evaluated in the current studies were all on the ascending limb of the dose-response curve to prevent any opioid-induced toxicity. Another caveat to be considered is the infusions of fentanyl that were delivered early in the session, when response costs were small, likely resulted in a less robust state of opioid withdrawal during the later portions of the session when determinations of reinforcing effectiveness (i.e., breakpoint) were being made. To circumvent any potential confounds of evaluating the effects of opioid withdrawal after delivery of multiple infusions of fentanyl, future studies will utilize demand curve analyses to evaluate the effects of morphine deprivation on elasticity of fentanyl demand. By increasing the ratio requirement across sessions, each ratio can be evaluated devoid of any “carry-over” effects (e.g., termination of withdrawal) of fentanyl infusions earned under smaller response requirements. Furthermore, quantifying the degree tolerance to the effects of fentanyl produced by this morphine dosing regimen (e.g., relative shifts in the reinforcing and antinociceptive effects of fentanyl) would further aid in interpretation of these data.

Given the prevalence of individuals abusing both opioids and stimulants, the current studies aimed to investigate whether morphine dependence and withdrawal would impact the reinforcing effectiveness of stimulants (e.g., methamphetamine and cocaine). The present data demonstrate that morphine dependence did not alter the reinforcing effectiveness of methamphetamine (0.032 mg/kg/infusion) or cocaine (0.32 mg/kg/infusion), relative to baseline, regardless of whether rats were in a state of morphine dependence or withdrawal. Indeed, few studies have demonstrated that acute administration of morphine and methamphetamine produces enhanced locomotor effects and toxicity relative to either drug alone (Namiki et al., 2005; Ito et al., 2007; Trujillo et al., 2011; Kaka et al., 2014), however, none of these studies directly assessed whether the reinforcing effectiveness of stimulants, such as cocaine and methamphetamine, are impacted by states of opioid dependence and withdrawal. Furthermore, that the reinforcing effectiveness of cocaine is unaffected by morphine dependence or withdrawal is consistent with previous studies that demonstrated that opioid dependence and withdrawal does not change the self-administration of a range of cocaine doses under fixed ratio schedules of reinforcement in rats (Leri et al., 2006; Cooper et al., 2010). It should be noted, however, that other studies suggest that morphine withdrawal can enhance the reinforcing effectiveness of cocaine when assessed under a progressive ratio schedule of reinforcement in rats (He and Grasing, 2004). One possible explanation for this difference is that the current studies used an acute-withdrawal procedure (20 h since previous morphine injection), whereas He and Grasing used both a larger dose of morphine (50 mg/kg/day) and a longer period of withdrawal (at least 5 days since previous morphine injection) prior to evaluating cocaine self-administration. In addition, although the current studies terminated PR sessions following a 45 min limited hold, He and Grasing terminated PR sessions after 3 h, independent of whether rats were still responding or not. In the current studies, sessions were typically terminated after 1–2 h. Taken together with the present data, these findings suggest that unlike when opioids are available for self-administration, the reinforcing effects of stimulants are largely unaffected by states of opioid dependence or withdrawal, likely contributing to the high prevalence of co-abuse of stimulants in opioid-dependent individuals.

In summary, the present data demonstrate that state of opioid dependence can impact the reinforcing effectiveness of opioids, with the emergence of opioid withdrawal driving increases in the reinforcing effectiveness of opioids, such as fentanyl. Furthermore, these data show that the state of opioid dependence has little effect on the reinforcing effectiveness of methamphetamine or cocaine. Taken together, these results suggest that development of novel treatment strategies are necessary to better treat individuals with co-occurring substance use disorders.

## Data Availability

The raw data supporting the conclusions of this article will be made available by the authors, without undue reservation.
